# Traitement préventif intermittent à la sulfadoxine – pyriméthamine du paludisme chez les femmes enceintes: efficacité et observance dans deux hôpitaux urbains du Burkina Faso

**DOI:** 10.11604/pamj.2013.14.105.1331

**Published:** 2013-03-17

**Authors:** Sanata Bamba, Adama Séré, Rodrigues Nikiéma, Tinto Halidou, Blandine Thiéba, Blami Dao, Robert Tinga Guiguemdé

**Affiliations:** 1 Laboratoire de Parasitologie-Mycologie, Institut supérieur des sciences de la santé, Université polytechnique de Bobo-Dioulasso, Burkina Faso; 2 Unité de formation et de recherche en sciences de la santé, Université de Ouagadougou, Burkina Faso; 3 Institut de recherche en sciences de la santé, Bobo –Dioulasso, Burkina Faso

**Keywords:** Paludisme, Femme, Grossesse, Traitement Préventif Intermittent, Sulfadoxine, Pyriméthamine, Efficacité, Observance, Burkina Faso, Malaria, woman, Grossesse, Intermittent Preventive Treatment, sulfadoxine, pyrimethamine, effectiveness, observance, Burkina Faso

## Abstract

**Introduction:**

La présente étude prospective se propose dévaluer l'efficacité thérapeutique du traitement préventif intermittent à la sulfadoxine - pyriméthamine et son observance chez la femme enceinte dans deux hôpitaux urbains au Burkina Faso.

**Méthodes:**

Chaque femme répondant aux critères d'inclusion a été soumise à un questionnaire pour la collecte des données socio - démographiques et des renseignements sur la grossesse. A l'accouchement, une apposition placentaire a été réalisée systématiquement. La lecture a été faite au microscope à lobjectif 100 à immersion.

**Résultats:**

Au total, 542 femmes ont été incluses avec un âge moyen de 26,0 ± 6,45 ans (extrêmes 13- 43 ans). Le taux de couverture du TPI à la sulfadoxine- pyriméthamine a été de 80%. Le taux d'infestation placentaire a été de 4,7%. Il a diminué avec le nombre de dose de traitement préventif intermittent. Il a augmenté cependant de juillet à octobre. De 42,9% en octobre, il a diminué significativement à 9,5% en novembre (p < 0,05). Le taux global de bonne d'observance a été de 55%. Il a augmenté avec l’âge (p < 0,05).

**Conclusion:**

Le taux de couverture de la sulfadoxine - pyriméthamine a été de 80%. Ce résultat est en conformité avec les objectifs du plan stratégique 2006-2010 de lutte contre le paludisme au Burkina Faso, qui préconisait un taux de couverture en sulfadoxine - pyriméthamine de 80% pour 2010. L'augmentation de la fréquence d'infestation de juillet à octobre, serait liée à la recrudescence de la transmission palustre pendant la saison des pluies (mai-octobre).

## Introduction

Chaque année, près de 25 millions de femmes enceintes, dont 20% de primipares, sont confrontées aux conséquences du paludisme en Afrique subsaharienne [1]. Le placenta en effet est le site préférentiel de séquestration et de développement des plasmodiums pendant la grossesse. La femme enceinte a ainsi une forte susceptibilité d’être infestée par *Plasmodium falciparum*. Ceci se traduit par une forte fréquente d’épisodes palustres avec une forte densité parasitaire par rapport aux femmes non enceintes [2]. L'infection palustre chez la femme enceinte se caractérise par une anémie maternelle. Les carences nutritives qui en résultent pour le foetus sont parfois source d'avortements, de retard intra utérine, de mort in utero et de prématurité [3, 4]. Au regard des conséquences du paludisme durant la grossesse, il est important que les femmes enceintes vivant dans les zones d'endémie palustre soient sous prophylaxie antipalustre [5].

En Afrique subsaharienne, l'OMS recommande des stratégies de prévention pendant la grossesse, basées sur l′administration d'un traitement préventif intermittent (TPI) et sur l′utilisation de moustiquaires imprégnées d′insecticide (MII) [6]. L'efficacité du TPI à la sulfadoxine-pyriméthamine (SP) a été démontrée en Afrique de l'Ouest [7–11] et en Afrique de l'Est [12, 13].

Au Burkina Faso, la prévention du paludisme chez la femme enceinte constitue une priorité pour les autorités sanitaires nationales. C'est ainsi qu'avec la chloroquino-résistance ayant atteint 26,9% à 63,3% en 2003 [14], une nouvelle stratégie de prévention du paludisme chez la femme enceinte a été mise en place à partir de Février 2005 [15].

Conformément aux recommandations de l'Organisation Mondiale de la Santé (OMS), cette stratégie préconise l'administration de la sulfadoxine-pyriméthamine (SP) en traitement préventif intermittent (TPI) chez la femme enceinte [15–17]. La thérapie intermittente pour le paludisme avec la SP est recommandée pour les femmes enceintes vivant en zone d'endémie palustre où *Plasmodium falciparum* est résistant à la chloroquine (CQ) et sensible à la SP [18, 19]. Le TPI avec au moins de deux doses de sulfadoxine - pyrimethamine administrées pendant le second et le troisième trimestre de grossesse représente une stratégie de prévention alternative dont l'efficacité a été démontrée par plusieurs études conduites en Afrique dans la réduction du taux de l'infestation placentaire par *Plasmodium falciparum* [7, 10, 18, 20–22], le faible poids de naissance [4, 19] et l'anémie sévère au cours de la grossesse [4, 23].

La prise de SP doit être observée par un agent qualifié au niveau de la formation sanitaire. Toutefois, le succès d'un tel programme de prévention dépend non seulement de l'efficacité du médicament utilisé et de sa disponibilité mais également de l'observance des directives par les femmes enceintes et les agents de santé. Depuis la mise en place de cette nouvelle politique au Burkina Faso, aucune donnée relative à l'efficacité du TPI à la SP chez la femme enceinte en milieu urbain n'est disponible [15, 16]. C'est dans ce contexte que la présente étude se propose trois années après l'adoption de cette stratégie, d’évaluer son efficacité thérapeutique et son observance chez la femme enceinte dans deux hôpitaux urbains du Burkina Faso.

## Méthodes

### Site d’étude

L’étude s'est déroulée dans les services de gynécologie-obstétrique du Centre Hospitalo-universitaire (CHU) de Ouagadougou et de Bobo - Dioulasso, les deux principales villes du Burkina Faso.

Le Burkina Faso est un pays sub-saharien situé dans la boucle du fleuve Niger et couvre une superficie de 274 200 Km^2^. Il possède un climat de type soudano-sahélien caractérisé par l′alternance d′une saison des pluies (mai-octobre) et d′une saison sèche (novembre-avril). Il existe une recrudescence de la transmission palustre pendant la saison des pluies à Ouagadougou et Bobo - Dioulasso.

### Population d’étude

Nous avons conduit une étude transversale prospective recrutant des femmes enceintes venues accoucher dans les services de gynécologie-obstétrique du CHU Yalgado Ouédraogo (CHUYO) de Ouagadougou et du CHU Sourô Sanou (CHUSS) de Bobo - Dioulasso entre février et novembre 2008.

Ont été inclues dans l’étude les femmes enceintes résidant dans les villes de Ouagadougou et de Bobo- Dioulasso et consentantes librement de participer à l’étude. Ces femmes ont au préalable bénéficié de consultations prénatales (CPN) trimestrielles dans les centres de santé et de promotion sociale (CSPS) ou dans les services de santé maternelle et infantile (SMI) des deux villes.

Les critères de non-inclusion dans l’étude ont été les suivants: les femmes en mauvais état général; celles porteuses de grossesses pathologiques pouvant altérer le placenta (hématome rétro-placentaire, placenta *praevia*) et les femmes qui ont affirmé avoir présenté une allergie à la SP et donc n'ayant pas respecté le TPI.

### Chimioprophylaxie

Comme le préconise le programme national de lutte contre le paludisme (PNLP) au Burkina Faso [16], la SP a été administrée à la posologie de 3 comprimés en prise unique (soit 1,5 g de sulfadoxine et 0,75 g de pyriméthamine). Cette administration a été faite lors des CPN conjointement à du fer et à de l'acide folique. Deux doses ont été en principe délivrées, l'une lors du second trimestre de la grossesse et la deuxième au troisième trimestre selon les recommandations de l'OMS [1].

### Collecte des données

Chaque femme répondant aux critères d'inclusion de l’étude a été soumise à un questionnaire après l'obtention de son consentement éclairé. Ce questionnaire a pris en compte les variables suivantes: l'identité et l’état civil de la femme, les antécédents obstétricaux (gestité), les conditions socio-économiques, les conditions socio-culturelles (religion, profession), le niveau d'instruction (analphabète, niveau d'instruction primaire, secondaire, ou supérieur), les particularités de la grossesse actuelle et les conditions d'observance de la chimioprophylaxie (prescription du TPI, nombre de prises, effets secondaires du TPI, autre chimioprophylaxie prescrite). Les carnets de santé des femmes ont été consultés afin de s'assurer des dates et lieux des CPN fréquentés, de la prescription de la SP ou d'une autre alternative, de la prescription d'autres traitements éventuels. Le degré de concordance entre les recommandations du médecin et les comportements de la femme a été vérifié. Les réponses de la femme ont été également comparées à celles mentionnées dans le carnet (chimioprophylaxie prescrite, nombre de prise, les antécédents obstétricaux).

### Diagnostic parasitologique

Lors de l'accouchement, l'apposition placentaire a été systématiquement pratiquée après obtention de l'autorisation de la femme ou des accompagnants. Des morceaux de la face maternelle du placenta ont été déposés sur des lames de façon à confectionner des frottis minces. La lecture a été faite au microscope à l'objectif 100 à immersion. Au total, 100 champs microscopiques ont été lus à la recherche de trophozoïtes ou de schizontes de plasmodiums témoins d'une infection palustre placentaire.

Il a été estimé à 200 en moyenne le nombre de globules rouges parasités par champ examiné, et à 4.000.000 le nombre de globules rouges par microlitre (µl) de sang. La densité parasitaire a été exprimée en nombre de parasites par microlitre de sang [24].

### Critères de jugement de l'efficacité et de l'observance

Le traitement à la SP a été considéré comme efficace en l'absence de *Plasmodium sp* à l'examen du placenta [1]. Il a été considéré qu'une femme a veillé à la bonne observance du traitement quand elle a reçu 3 comprimés de SP en une prise orale au deuxième trimestre et au troisième trimestre [16].

### Considérations éthiques

Avant le début de l’étude, son protocole a été soumis et approuvé par le comité d’éthique institutionnel du Centre Muraz à Bobo-Dioulasso au Burkina Faso.

### Analyse des données

Les données ont été analysées sur le logiciel EPI-INFO version 6.04. Le test statistique de Chi carré au seuil de signification de 5% a été utilisé pour la comparaison des proportions.

## Résultats

Au total, 542 femmes ont participé à l’étude. Leur âge moyen a été de 26,0 ± 6,45 ans (extrêmes 13-43 ans). Les femmes de moins de 18 ans ont représenté 26,6% (n = 144) vs 73,4% (n = 398) pour celles de plus de 18 ans (Tableau 1), (p<,05). Les femmes analphabètes ont représenté 41,5% (n= 225) vs 58,5% (n= 159) de scolarisées (Tableau 1), (p >0,05). Une analyse des caractéristiques culturelle et professionnelle de la population d’étude indique que parmi les 542 femmes, les ménagères ont constitué 50,5%( n= 273), ont suivi ensuite les commerçantes (24,3%, n= 132 /542) puis les élèves-étudiantes (17,3% n= 94) et enfin les fonctionnaires (8%, n= 43) (Tableau 1), (p > 0,05). La religion musulmane a été pratiquée par 56,7% (n= 307) des femmes; vs 44,3% (n= 235) de chrétiennes (Tableau 1), (p > 0,05).

**Tableau 1:** Caractéristiques socio – démographiques et taux de bonne observance du TPI à la SP

Caractéristiques	Effectifs global (N = 542)	Effectif sous TPI à la SP (N = 433)	Taux de bonne Observance (N= 238)
**Age**			
< 18 ans	144 (26,6%)	89 (61,8%)	19 (21,3%)
>18 ans	398 (73,4%)	354 (90%)	219 (61,9%)
**Niveau d'instruction**			
Analphabète	225 (41,5%)	171 (76%)	52(30,4%)
Primaire	157 (29%)	132 (84%)	59 (44,7%)
Secondaire	111 (20,5%)	102 (91,9%)	91 (89,2%)
Supérieur	49 (9%)	38 (77,5%)	36 (94,7%)
**Profession /occupation**			
Ménagère	273 (50,5%)	240	147 (61,1%)
Commerçante	132 (24,3%)	92	48 (52,2%)
élèves-étudiante	94 (17,3%)	83	35 (42,1%)
Fonctionnaires	43 (8%)	28	8 (28,5%)
**Religion**			
Musulmane	307 (56,7%)	237 (77,2%)	129 (54,3%)
Chrétienne	235 (44,3%)	196 (83,4%)	109 (55,6%)
**Antécédents obstétricaux**			
Gestité			
Primigeste	220 (40,6%)	183 (42,3%)	81 (43,3%)
Secondigestes	135 (24,4%)	98 (22,6%)	53 (51,4%)
3 et plus	187 (34,5%)	152 (35,1%)	104 (68%)
**Gratitude de la SP**			
SP gratuite	-	96 (21,7%)	51 (53,1%)
SP achetée	-	347(78,3%)	187 (54%)
**Effets secondaires à la SP**			
Effets secondaires notifiés	-	88 (19,9%)	46 (52,3%)
Effets secondaires non notifiés	-	355 (80,1%)	192 (54,1%)

TPI : Traitement préventif intermittent ; SP : sulfadoxine – pyriméthamine

### Antécédents obstétricaux et type de chimioprophylaxie

Le nombre moyen de grossesses de la population d’étude a été de 2,7 avec un maximum de 11 grossesses. La proportion des primigestes, secondigestes et de multigestes ont été respectivement de 40,6% (n= 220), 24,9%(n= 135) et de 34,5% (n= 187) (Tableau 1), (p > 0,05).

Sur 542 femmes, la chimioprophylaxie prescrite au cours des CPN a été la SP pour 80% (n= 433) vs 8,7% (n= 47) pour la CQ en chimioprophylaxie hebdomadaire (25 mg/kg sur 3 jours à la première CPN, puis 300 mg/semaine) (Tableau 2). Et, 11,4% (n= 62) des femmes n'ont reçu aucune prophylaxie médicamenteuse antipalustre (Tableau 2). Parmi les 433 femmes qui ont été sous la chimioprophylaxie à la SP, la majorité ont reçu les deux doses (55% n= 238); vs 29,5% (n= 128); 13,1% (n= 57) et 1,2% (n= 5) pour respectivement une, trois et quatre doses. Les femmes ignorant le nombre de doses de SP reçues ont été au nombre de 5 (1,2%) (Tableau 2).

**Tableau 2:** Répartition des femmes selon la chimioprophylaxie et le nombre de dose de SP

Nombre de prise de SP prescrites	Effectif	%
**Chimioprophylaxie**		
SP	433	80%
Chloroquine	47	8,7%
Aucune chimioprophylaxie	62	11,4%
**Nombre de prise de SP**		
1	128	29,5%
2	238	55%
3	57	13,2%
4	5	1,2%
**Inconnu**	5	1,2
**Total**	433	100

SP: sulfadoxine – pyriméthamine

L'analyse des antécédents obstétricaux indique que parmi les 433 femmes ayant reçu le TPI à la SP, 42,3% (n= 183), 22,6% (n= 98), et 35,1% (n= 152) ont été respectivement primigestes, secondigestes et multigestes (Tableau 1). Selon la gratuité de la SP, 78,3% (n= 347) des femmes ont affirmé avoir acheté la SP, vs 21,7% (n= 96) qui l'ont reçu gratuitement (Tableau 1).

### Influence de la chimioprophylaxie à la SP sur l'infestation palustre placentaire

De façon globale, sur 443 appositions placentaires analysées, 4,8% (n = 21) ont eu des trophozoïtes de *Plasmodium falciparum*. Le taux d'infestation placentaire à *Plasmodium falciparum* a été de 7% (9/128), 4,2% (10/238) et 3,5% (2/57) pour une, deux et trois doses respectivement (Tableau 3). Aucune relation entre le nombre de dose et le taux d'infestation placentaire n'a été établie. (p > 0,05).

**Tableau 3:** Efficacité du TPI à la SP selon le nombre de grossesse et de dose de SP

Variables liées à l'infestation placentaire	Placenta		
	Positif	Négatif	Total
**Nombre de geste**			
Primigestes	7 (3,7%)	180	187
Secondigestes	9 (8,7%)	94	103
Multigestes	5 (3,5%)	148	153
Total	**21(4,7%)**	**422**	**443**
**Nombre de dose**			
1	9 (7%)	119	128
2	10 (4,2%)	228	238
3	2 (3,5%)	54	57
**Autre**	0	10	10
**Total**	**21 (4,7%)**	**401**	**433**

TPI: Traitement préventif intermittent; SP : sulfadoxine – pyriméthamine

### Relation entre le nombre de grossesses et l'infestation placentaire

Parmi les 21 (4,8%) de frottis placentaires qui ont eu des plasmodiums, les primigestes et les secondigestes ont représenté 76,1% (16/21). Le taux d'infestation placentaire a été plus élevé chez les secondigestes (8,7; 9/103) que chez les primigestes (3,7%; 7/187) (Tableau 3). Toutefois, ces différences n'ont pas été statistiquement significatives (p > 0,05).

**Figure 1:**
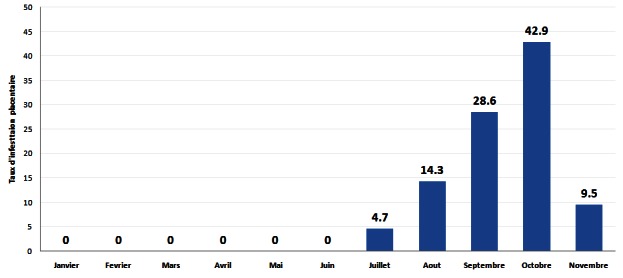
Evolution du taux d'infestation placentaire

En analysant la répartition temporelle de l'infection placentaire, il ressort qu'aucune infestation palustre placentaire n'a été observée durant les 5 premiers mois de l’étude (février à juin). Le taux d'infection placentaire a augmenté de juillet à octobre (42,9%, 9/21) et a diminué significativement à 9,5% (2/21) en novembre. Nous avons pu établir une relation entre la répartition temporelle de l'infection placentaire (p < 0,05) (Figure 1).

### Observance du TPI à la SP

Le taux global de bonne observance du TPI à la SP a été de 55% (238/433). Nous avons pu établir une relation entre la bonne observance du TPI et l’âge des femmes. En effet, le taux de bonne observance a été de 21,3% (19/89) chez les femmes de moins de 18 ans et de 61,9% (219/354) chez les plus de 18 ans (Tableau 1), (p 0 < 0,05).

En analysant la relation entre cette bonne observance et le niveau d'instruction, il ressort que le taux de bonne observance a été de 94,7% (36/38), 89,2% (91/102), 44,7% (59/132) et 30,4% (52/171) pour les femmes du niveau d'instruction supérieur, secondaire, primaire et les analphabètes, respectivement (Tableau 1). Nous n'avons pas pu établir une relation entre la bonne observance et le niveau d'instruction (p > 0,05).

Nous n'avons pas noté de relation entre l'observance et la confession religieuse (Tableau 1). En effet, le traitement a été bien observé aussi bien chez les femmes chrétiennes (55,6%; 109/196) que chez les femmes musulmanes (54,3%; 129/237), (p > 0,05).

La meilleure observance de 61,1% (147/240) a été notée chez les ménagères. Les niveaux d'observance ont diminué ensuite, avec des taux de 52,2% (48/92 pour les commerçantes, 42,1% (35/83) pour les élèves - étudiants et 28,5% (8/28) pour les fonctionnaires (Tableau 1). Cependant nous n'avons pas trouvé de relation entre l'observance et la profession des femmes (p >0,05).

Le taux de bonne observance a été de 36,6% (67/183) chez les primigestes, 44,3% (81/183) vs 54,1% (53/98) et 68,1% (104/152) pour les secondigestes et les multigestes (Tableau 1). Cependant nous n'avons pas trouvé de relation entre l'observance et le nombre de grossesses (p > 0,05).

L'observance a été similaire entre les femmes qui ont reçu la SP gratuitement (53,1% (51/96) et celles qui l'ont achetée (54%; 187/347). Cependant nous n'avons pas trouvé de relation entre l'observance et la religion des femmes (p >0,05) (Tableau 1).

L'observance a été bonne chez 52,3% (46/88) des femmes ayant déclaré avoir présenté un effet secondaire et de 54,1% (192/355) chez celles qui ne l'ont pas signalé (P > 0,05) (Tableau 1).

Une analyse de l'incidence de ces effets secondaires indique que 20,3% (88 /433) des femmes ont déclaré avoir présenté au moins un effet secondaire à la suite de la prise de la SP (Tableau 1). Les effets secondaires les plus fréquemment rapportés ont été l'asthénie (30,7%; 27/88); les vomissements (27,2%; 24/88); les nausées (239%; 21/88) et les sensations de vertiges (18,2%; 16/88) (Tableau 1). Tous ces effets ont survenu en moyenne dans les 4 heures suivant la prise médicamenteuse.

## Discussion

L'efficacité du TPI à la SP a été démontrée en milieu rural du Burkina Faso à travers trois études [7, 20, 21]. Ces études ont rapporté une bonne efficacité de cette stratégie à travers une réduction de la charge parasitaire placentaire palustre chez la femme enceinte. Cependant il n'existe aucune donnée en ce qui concerne le milieu urbain, qui pourtant présente un faciès complètement différent du point de vue épidémiologique et socio- culturel. La présente étude qui est la première réalisée en milieu urbain permettra de fournir au PNLP des données complémentaires sur les deux villes (Ouagadougou et Bobo -Dioulasso) les plus peuplées du Burkina Faso. L’âge moyen des parturientes est de 26 ± 6,45 ans, chiffre voisin de celui d'une étude menée en milieu rural au Burkina Faso [20], au Bénin [25] et au Nigeria [11].

Dans la présente étude, plus de la moitié des femmes (80%) ont bénéficié du traitement prophylactique tel que préconisé par le PNLP, la majorité d'entre elles (55%), ont reçu deux doses de SP (Tableau 2). Le même constat a été fait par les travaux antérieurs conduits au Burkina Faso (96,2%) [7]. Au vu de cette bonne proportion, nous pensons que la sensibilisation des femmes à la problématique du paludisme gestationnel initiée à travers les campagnes d'information, d’éducation et de communication (IEC) du PNLP a un impact sur l'adhésion des femmes enceintes au TPI à la SP au Burkina Faso [16]. Des taux similaires au nôtre ont été rapportés ailleurs au Ghana (77%) [10].

Par ailleurs, dans la présente étude, les femmes ayant bénéficié d'une chimioprophylaxie par la chloroquine ont représenté 8,7% de notre population d’étude. Bien que l'objectif de notre étude n’était pas de comparer les deux médicaments (SP et CQ), nos résultats indiquent que trois ans après la mise en place de la nouvelle politique de prévention du paludisme, et malgré le retrait de la chloroquine, celle-ci est toujours utilisée pour la prévention du paludisme chez la femme enceinte. Bien que cette utilisation ne concerne qu'une faible proportion de notre population d’étude (8,7%), il soulève néanmoins la question de la problématique du changement de comportement et de l'appropriation par les praticiens et les populations des nouvelles stratégies d'intervention en matière de santé publique. Toutefois, les résultats obtenus ont le bénéfice de confirmer que le TPI à la SP est efficace en chimioprophylaxie antipalustre chez la femme enceinte, comme cela avait été précédemment démontré par des études antérieures conduites au Burkina Faso [7, 20, 21], et suggéré par le Ministère de la santé du pays [16].

Le taux d'infestation placentaire palustre global de la présente étude est de 4,7% (Tableau 3) chez des femmes ayant bénéficié du TPI à la SP. Des taux similaires au nôtre ont été enregistrés au Bénin (4,1%) [25]. Par contre des taux supérieurs au nôtre (4,7%) ont été rapportés par d'autres auteurs. Ces taux ont été en effet de 19,2% au Burkina Faso selon une étude antérieure [21]. Ailleurs au Mali, des taux de 16,7% [26], à 24,5% [8] ont été notifiés par des études. Les mêmes tendances de 13,8% et de 22,8% ont été respectivement rapportées au Kenya [13] et au Malawi [27]. Le faible taux observé dans notre étude comparé à celui des études antérieures menées au Burkina Faso [21] pourrait s'expliquer par la bonne observance globale observée dans notre étude. Toutefois de façon générale, ces constats renforcent l'efficacité de la SP en chimioprophylaxie antipalustre pendant la grossesse et justifie de ce fait son administration en traitement préventif intermittent (TPI) depuis février 2005 au Burkina Faso [16].

La thérapie intermittente à la SP est efficace et peut être utilisée comme une stratégie pratique pour la réduction du risque de l'infestation placentaire par des plasmodiums en zone d'endémie palustre. Les mêmes conclusions ont été faites par des auteurs au Burkina Faso [7, 20, 21], ailleurs en Afrique de l'Ouest au Mali [8, 26], au Bénin [25], au Nigéria [11], au Ghana [10], et d'autre part en Afrique de l'Est au Kenya [14] et au Malawi [18, 27].

Par ailleurs, les primigestes et les secondigestes sous TPI à la SP demeurent les plus exposées au paludisme, 76,1% d'entre elles ont eu, en effet, un frottis placentaire positif (Tableau 3). Les mêmes tendances ont été observées au Burkina Faso [21], au Bénin [25] et au Malawi [18, 27]. Cela pourrait s'expliquer par le fait que les multigestes, développent avec les grossesses antérieures une immunité protectrice contre l'infection palustre placentaire [28, 29].

Le fait qu'aucun placenta n'est été trouvé parasité durant les 5 premiers mois de l’étude, puis que la fréquence d'infestation ait augmenté de juillet à octobre, est lié à la recrudescence de la transmission palustre pendant la saison des pluies (mai-octobre) (Figure 1). Le même constat a été fait en milieu rural au Burkina Faso par des auteurs qui ont noté que 90,6% de la prévalence du paludisme placentaire était notée pendant la saison pluvieuse [21]. Cela devrait interpeller les autorités sanitaires sur la nécessité de développer des stratégies de surveillance rapprochée pour une meilleure observance du TPI pendant la saison de transmission où le risque est plus important.

Le taux de couverture du TPI à la SP des femmes de notre étude a été de 80%. Ce résultat est en conformité avec les objectifs du plan stratégique 2006-2010 de lutte contre le paludisme au Burkina Faso, qui préconisait un taux de couverture en SP de 80% pour 2010 [16].

Le taux d'ensemble de bonne observance du traitement à la SP, de 55%, est bien inférieur à celui de 99,1% dont fait état une étude menée au Mali [8]. Si dans notre étude, la gratuité de la SP à 78,3% ne semble avoir eu d'influence sur l'observance, dans l’étude réalisée au Mali [8], chaque femme recevait gratuitement la SP lors des consultations prénatales et cela a semblé jouer positivement sur le taux de couverture.

Des études complémentaires devraient être conduites dans notre cas pour comprendre les raisons du taux de 55% malgré la gratuité chez 78,3% des femmes. Des raisons de nature socioculturelle, économique et environnementale pourraient en être les causes.

L'analyse des facteurs socio-démographiques fait apparaître que l’âge, et non la gestité, constitue un déterminant majeur de l'adhésion au TPI. Nous avons, en effet, observé une amélioration de l'observance avec l’âge des femmes. Cela pourrait être lié à une meilleure prise de conscience des complications du paludisme au cours de la grossesse.

## Conclusion

Notre étude, qui est la première réalisée sur l'efficacité du TPI en milieu urbain au Burkina Faso, confirme l'efficacité plusieurs fois rapportée de cette stratégie lorsqu'elle est bien suivie. Cette étude qui confirme également l'efficacité du TPI à la SP et aussi la prescription de la chloroquine en chimioprophylaxie, soulève cependant la question de l'appropriation des nouvelles stratégies d'intervention en santé publique. En effet, 3 ans après le changement de la politique et le retrait de la chloroquine, cette dernière est toujours prescrite et utilisée. Avec un taux de couverture du TPI à la SP de 80%, les résultats de notre étude confirment l'atteinte en milieu urbain des objectifs du plan stratégique 2006-2010 de lutte contre le paludisme au Burkina Faso, qui préconisait un taux de couverture en SP de 80% pour 2010. Cependant les raisons du taux d'observance de 55%, malgré la gratuité de la SP chez 78,3% des femmes de notre série dans certains cas méritent d’être explorées en tenant compte des facteurs socioculturels, économiques et environnementaux de notre contexte d’étude.
